# Plastic Gold

**Published:** 1868-07

**Authors:** John G. Mackintosh


### ARTICLE III.

### *Plastic Gold.*

By John G. Mackintosh, D. D. S.

Plastic gold has now been used by the profession for some years, and while in many cases it has been found to answer a good purpose, in others repeated failures have led to its abandonment. The object of the writer of this article is to lay before the readers of the Journal some ideas gained by several years of experience in the use of this preparation of gold for filling teeth.

Of the many forms of plastic gold, several of them if properly manipulated, will be found truly serviceable in producing a durable as well as a beautiful filling; the greatest objection which can be urged against them being a want of uniformity occasioned by a defect in the manufacture.

The bright, shred-like masses only are fit for use, and all portions having a dull, or dark-brown color, with a tendency to crumble, should be rejected. It frequently happens that one portion of the mass contained in a box, consists of a good quality, while another portion of the same box is very inferior; the inferior portion such as has been described as being of a dull or dark brown color, however, may be used in the bottom of large cavities, (grinding surface cavities for example) which, from their shape, are well adapted for gold in any form.

After selecting the proper quality of plastic gold, prior to introducing it into the cavity, it should be annealed, by tearing from the mass small portions of a size suitable for the cavity to be filled, and placing these in the dish of an alcohol annealing lamp.

When the gold is thus prepared the next step is to protect the cavity from all moisture, it being far from the truth that this form of gold can be introduced under water as well as above, or in a dry state.

Perfect dryness is very essential in manipulating this form of gold, and many fail on account of their carelessness in this respect, or through ignorance of the means by which it can be accomplished.

The proper application of the "rubber dam" will in every case ensure the dryness of the cavity, but as some patients may object to the use of this appliance, I will take occasion to describe a method by which I succeed in accomplishing the purpose, and save some of the time which is consumed in adjusting the sheet of rubber: A napkin some twelve or fourteen inches long by six or eight inches wide, is split at one end for a distance of four or five inches so as to form two tails. One of these tails is folded into a strip of an inch and a half in width, which, after drying the mucous membrane, is placed over the labial surface of the gum (if the cavity or cavities to be filled are upon the approximal surfaces of the superior front teeth.) The same form is given to the other tail of the napkin, and it is placed over the palatine surface of the gum, the tooth being secured between the two tails, which are kept in place by the thumb and fore finger of the left hand of the operator.

The free end of the napkin may be used to secure the mouth of a duct, should this be necessary. Before applying the tails of the napkin, and after wiping dry the mucous membrane, small oblong folds of bibulous paper should be placed on the gum and the tails of the napkin applied over these. The napkin being in place, the cavity is first dried with cotton and afterwards with bibulous paper; then a twisted roll of bibulous paper is pressed between the approximal surfaces of the teeth and forced up, by gentle pressure, under the free edge of the gum beyond the cavity to be filled. If the adjoining tooth has been lost, the ends of the roll of bibulous paper, which project beyond the approximal surfaces, can be secured by the fingers holding the napkin in place. By such simple means as I have described, cavities upon the approximal surfaces can be effectually protected, and by a slight modification, this method can be adapted to cavities upon other surfaces besides the approximal ones.

The cavity being now ready to receive the gold, which, by the time the napkin is secured, has become sufficiently annealed, small masses are introduced against the cervical wall and well condensed, allowing the ones near the orifice of the cavity to project a short distance beyond the margin. The gold is introduced mass after mass, by means of serrated instruments of such a shape as will carry them well against the walls, until one-third of the cavity is filled from this direction. When this is done I commence introducing the masses against the wall next to the cutting edge of the tooth, and fill from this point again towards the centre of the cavity, taking care from the first to carry the gold well against the labial and palatine walls at the same time that I am building from the cervical and cutting-edge walls. I have at length but a simple cavity left in the centre of the filling, into which the gold is introduced in the same manner. By pursuing such a method as I have described, I do not at any stage of the operation depend wholly upon the adhesive property of the gold although I take advantage of this property also, but wedge the gold in.

If, from any cause, one mass of gold does not weld to another beneath it, it is still held as firmly in place as when plain gold in the form of the strip or rope is used. But should I build from the bottom of the cavity to the orifice, one layer upon another, as many do in using plastic gold, any failure of cohesion between these layers would endanger the filling. Where the cavity is one of considerable depth an small in diameter, I build layer upon layer from the bottom to within one third of the orifice and fill the remaining part as before described. In many cases it is advisable to finish the surface of a filling composed of plastic gold, with adhesive foil in the form of pellets cut from a rope; and in all cases where I build up part of the crown in order to restore the form of the tooth, I prefer the adhesive foil to any of the plastic forms. I have observed, in my experience with plastic gold, that considerable care is necessary in finishing up that portion of the surface about the margin of a filling wholly composed of this form of gold, there being a brittleness somewhat similar to that possessed by cast iron, while the adhesive and soft foils resemble malleable or wrought iron. It is therefore well not to build the gold high above the orifice of a cavity, and to use a fine file in cutting down the surface; advantage is also derived from the use of the burnisher before filing, and during the process of cutting down the gold to a line with the orifice of the cavity.

---

